# Improving computer vision for plant pathology through advanced training techniques

**DOI:** 10.1002/aps3.70010

**Published:** 2025-06-07

**Authors:** Jamie R. Sykes, Katherine J. Denby, Daniel W. Franks

**Affiliations:** ^1^ Department of Computer Science University of York, Deramore Lane York YO10 5GH Yorkshire United Kingdom; ^2^ Centre for Novel Agricultural Products, Department of Biology University of York, Wentworth Way York YO10 5DD Yorkshire United Kingdom; ^3^ Department of Biology University of York, Wentworth Way York YO10 5DD Yorkshire United Kingdom

**Keywords:** computer vision, disease detection, machine learning, semi‐supervised learning

## Abstract

**Premise:**

This study investigates advanced training techniques to improve the performance of convolutional neural networks for disease detection in cocoa, *Theobroma cacao*.

**Methods:**

Despite recent stagnation in accuracy improvements in computer vision for image classification, our research demonstrates significant advancements in performance through semi‐supervised learning, specialised loss functions, and the inclusion of a non‐cocoa class.

**Results:**

Semi‐supervised learning reduced overfitting and enhanced generalisability, particularly for subtle symptoms. The non‐cocoa class exposed models to a broad range of relevant features, significantly improving model robustness and performance in difficult cases. Grad‐CAM for qualitative assessment provided valuable insights into model behaviour, highlighting cases of overfitting missed by summary statistics. We also describe dynamic focal loss, a novel loss function that uses an empirical measure of difficulty to weight each image. Our results suggest that while PhytNet shows promise in terms of computational efficiency and superior handling of difficult images, ResNet18 with semi‐supervised learning and dynamic focal loss emerged as the strongest contender for real‐world deployment.

**Discussion:**

This research underscores the potential of semi‐supervised learning and advanced loss functions in enhancing the applicability of deep learning models in agricultural disease management. It also presents a new high‐quality benchmark dataset of 7220 images of diseased and healthy cocoa trees, offering a much greater and more realistic challenge than the Plan Village dataset.

Major advances in the accuracy of computer vision (CV) classification models through architectural innovations have become less frequent in recent years. For example, the 60 million parameter ResNet152 (He et al., 2016), published in 2016, scores 82% accuracy on ImageNet (Deng et al., 2009), while ConvNeXt Large, published in 2022, scores 84% with 198 million parameters (Liu et al., 2022). Unfortunately, the large boost in performance seen in generative CV and language models that transformer architectures have facilitated does not seem to apply to image classification. While Vision Transformer (ViT) H14 pre‐trained with the Supervised Weakly through hashtAGs (SWAG) method (Singh et al., 2022) achieves 88.5% accuracy on ImageNet, it also has 634 million parameters and 1017 giga floating point operations (GFLOPS), making it too computationally expensive for many applications. The more sensibly scaled ViT B16, on the other hand, achieves 85% accuracy when pre‐trained using SWAG with 87 million parameters but only 81% accuracy when trained from random weights, as the above instances of ResNet152 and ConvNeXt were.

Despite this lack of notable improvement, there has been a plethora of new architectures published that claim to slightly improve the classification accuracy of models on benchmark datasets like ImageNet, without considering the potential negative implications of such a myopic focus on accuracy as a single performance metric. These include (1) the risk that models overfit to the specific characteristics of the training set that also exist in the test set because both subsets are sampled from the same distribution; (2) neglect of model explainability resulting from a lack of consideration of what data features the model is utilising to make predictions; (3) neglect of resource use efficiency with ever‐increasing model sizes using more computational power and electricity, especially when deployed at scale (e.g., OpenAI's ChatGPT [OpenAI, San Francisco, California, USA] presently uses about 500,000 kWh per day [Kolbert, 2024]); and (4) neglect of underrepresented clusters, groups, or populations in data, leading to poor generalisation and ethical concerns. A good example of this latter risk is the underrepresentation of indigenous groups in genomic sequence databases, which has led to misdiagnoses of genetic disorders and misrepresentation of genealogy (Morgan et al., 2019). This lack of diverse representation in data is also of concern in plant pathology, where a classifier may have 99% test set accuracy on common disease symptoms but be unable to generalise to newly evolved symptoms or less common symptoms resulting from uncommon environmental factors.

The aim of our initial paper introducing PhytNet (Sykes et al., 2025) was not necessarily to squeeze more accuracy from techniques or features like new layers for convolutional neural networks (CNNs) or transformers; instead, our goal was to ensure that model and dataset design choices suited the problem at hand and would promote generalisable and explicable model behaviour. Here, we look to further promote such favourable behaviour through improved data collection and advanced model training techniques. We do so using a novel image dataset of cocoa trees, *Theobroma cacao* L., that were healthy or infected with black pod rot (*Phytophthora palmivora* Butler [Pandian et al., 2021]), witches' broom disease (*Moniliophthora perniciosa* (Stahel) Aime & Phillips‐Mora [Aime and Phillips‐Mora, 2005]), or frosty pod rot (*Moniliophthora roreri* (Cif.) H.C. Evans, Stalpers, Samson & Benny [Evans et al., 1978]) at various stages of infection. This dataset (A) is a difficult challenge for image CV classification models, (B) is ideally suited to semi‐supervised learning, and (C) represents a serious real‐world problem causing economic hardship and environmental damage (Malhi et al., 2008; Kuok Ho and Yap, 2020). Semi‐supervised learning is a broad topic but essentially involves providing a model with labelled training data and allowing the model to autonomously add more examples to this training data as it learns.

SWAG is an alternative method to semi‐supervision. Here “weak supervision” is essentially a euphemism for poorly assigned labels and allows pre‐training on vast datasets scraped from the web with no labelling costs. The downside of this type of approach is that the model is treated like a black box, with any amount of overfitting accepted as long as the test set accuracy is improved. Such approaches to instructing statistical models come with great risk, as there is no telling what spurious lessons the model has learned from the data provided to it.

CV for plant pathology is now sufficiently performant that practitioners are deploying models in the real world with aims such as increasing pesticide use efficiency (Sapkota et al., 2023), managing international disease spread (Ulhaq et al., 2020), and improving crop yield (Tian et al., 2020). However, it is also now easier than ever to produce models with seemingly high test set accuracy that does not translate to real‐world efficacy (Sykes et al., 2024). This is due to (A) easy‐to‐use open source deep learning libraries like PyTorch (Paszke et al., 2019) and TensorFlow (Abadi et al., 2016), (B) cheap access to computing resources via platforms like Google Colab (Google, Mountain View, California, USA), and (C) free access to huge open source vision models like ConvNeXt (Liu et al., 2022) and ViT (Dosovitskiy et al., 2021). If not trained well, these advanced architectures can easily memorise the features of a dataset and so will not generalise well to the real world (Sykes et al., 2025).

A tool with strong potential for avoiding overfitting in these large neural networks is semi‐supervised learning—specifically, the wrapper method, which is considered one of the oldest forms of semi‐supervised learning (Zhu, 2008). This involves allowing a model to (1) learn from a small pre‐labelled set of data and then (2) iteratively and automatically label new data before recommencing training. Typically, the main focus of semi‐supervised methods is to leverage large amounts of unlabelled data given a modest amount of labelled data (Ouali et al., 2020; Van Engelen and Hoos, 2020). While this approach has proven effective at improving accuracy in some cases (Guillaumin et al., 2010; Yalniz et al., 2019), it has the potentially fatal flaw of risking contamination of the training data with incorrectly labelled observations from the unlabelled data. Here, we introduce a subtle augmentation to the wrapper method by applying it to a fully labelled dataset and having the model apply labels to progressively more difficult images that it has labelled correctly. This method provides multiple benefits: (1) it allows for learning from images without human‐visible disease symptoms that will only be added to the training set if the model detects relevant machine‐visible symptoms, (2) certainty is implicit so it does not require uncertainty estimation, and (3) it discourages runaway overfitting, where a model applies labels based on overfit features and then compounds this error by learning from that mislabelled data. However, this method does not allow for the benefit of learning from large amounts of unlabelled data.

Before the explosion in the use of deep neural networks, semi‐supervised learning had already been applied many times, and arguably to more suitable types of models. For example, when applied to generative mixture models, the ability of these models to estimate the uncertainty of predictions is invaluable in automatically assigning labels to data (Zhu, 2008). While the softmax of the logit values of a deep neural network can be used as a form of certainty estimate, it should be used with caution for real‐world applications due to issues arising from suboptimal calibration and out‐of‐distribution data (Pearce et al., 2021; Valdenegro‐Toro and Mori, 2022). As such, the application of semi‐supervised learning to this common form of deep learning is inherently disadvantaged. The estimation of uncertainty in neural networks is possible through methods like Bayesian neural networks and deep ensemble models (Valdenegro‐Toro and Mori, 2022), but this is beyond the scope of this discussion. Other successes in semi‐supervised learning include its use with graph‐based methods, where the nodes of a graph represent labelled and unlabelled observations, while the edges represent the similarity between observations (Zhu, 2008). This clustering‐type method is powerful because it allows for a continuous scale of similarity that is not allowed in the more rigid classification approach described here.

In this paper, we explore the efficacy and nuances of implementing techniques such as semi‐supervised learning, with a focus on discouraging overfitting and undesirable model behaviour while learning the more pervasive features of a dataset. We also look at other means to achieve these aims, such as a novel dynamic focal loss function (DFLoss) and additional classes in the dataset (n.b., A loss function is a mathematical function that quantifies the difference between the predicted output of a model and the actual target values, providing feedback to guide the learning process by minimising this difference). Finally, we address the critical task of model selection based on a comprehensive set of criteria. These include (1) summary statistics, (2) model attention and generalisation capabilities, and (3) runtime speed and computational requirements. This multifaceted evaluation approach promotes informed model choice for real‐world deployment.

## METHODS

### Data collection

Two datasets were used in this study: (1) a dataset of 57,228 healthy and diseased forestry and arable crop images scraped from Google and Bing, hereafter referred to as the FAIGB dataset (Sykes et al., 2024), and (2) a dataset of 7220 RGB images of diseased and healthy cocoa trees collected from across Ecuador and augmented with images from the web. The first dataset is described in detail by Sykes et al. (2024); we collected the novel second dataset for this study and will now describe it in detail.

**Figure 1:**
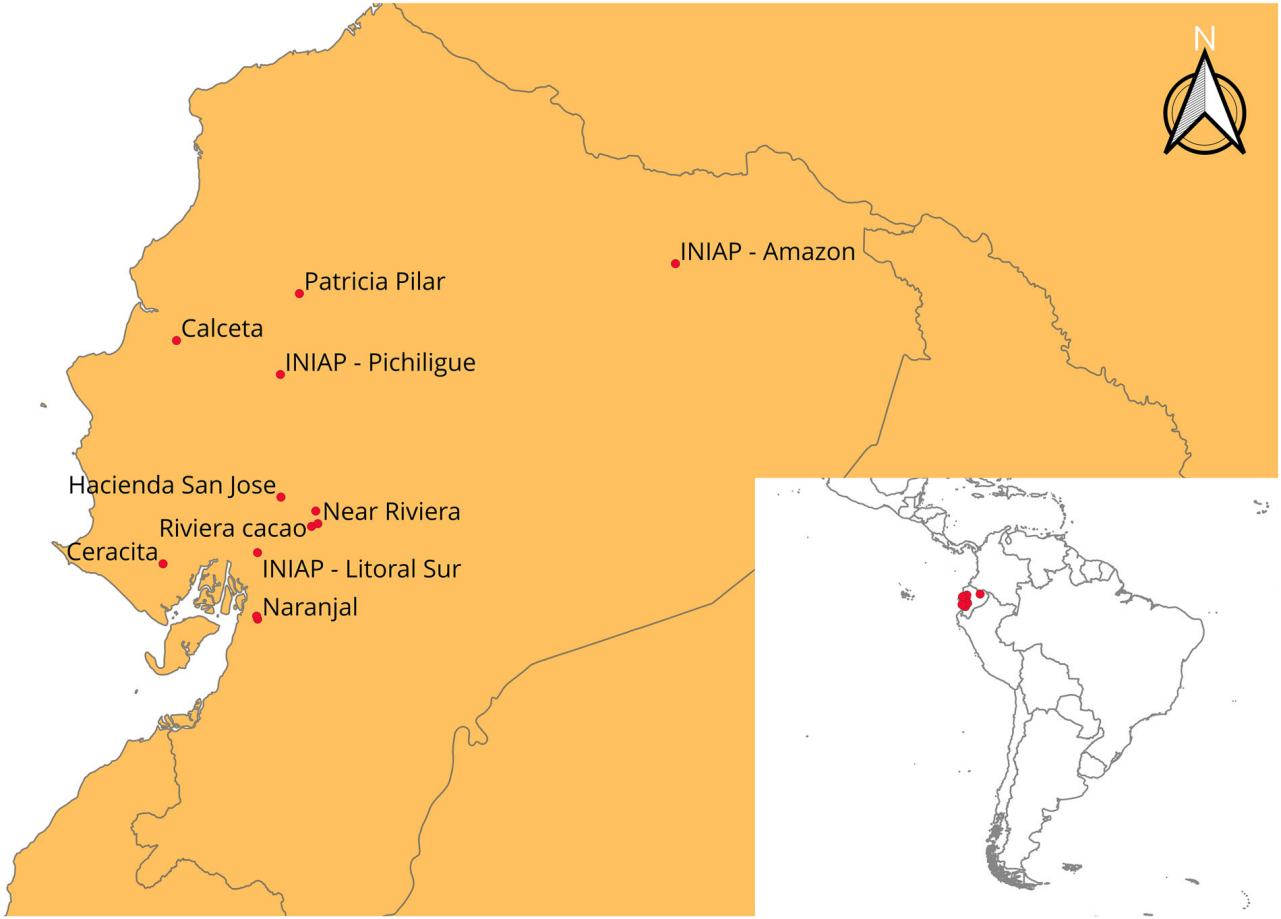
Farm and research station sites of cocoa image data collection across Ecuador. These sites span the three distinct cocoa growing regions of Ecuador: (1) the dry, west coastal region represented here by the two western‐most points (Ceracita and Calceta); (2) the humid and high‐yielding valley between the Cordillera Chongón‐Colonche and the Andes mountains, where the farms and research stations are represented by the eight centre points; and (3) the region between the Andes and the Amazon rainforest, known for high yields and genetic diversity of cocoa trees, where the research station (INIAP Amazon) is represented by the eastmost point.

The locations of the sites where these images were gathered cover three distinct growing regions (Figure 1, Appendix 1). The two locations west of the Cordillera Chongón‐Colonche (Ceracita and Calceta) represent the coastal region of Ecuador where cocoa crop yields are smaller but the trees are less prone to attack by fungal or oomycete disease because of the dry conditions. Between the Cordillera Chongón‐Colonche and the Andes is an area known for high‐quality and high‐yield cocoa production but with high rainfall and humidity, which encourages rampant disease. The third area, represented here by the Instituto Nacional de Investigaciones Agropecuarias (INIAP) Amazon research station, is east of the Andes and west of the Amazon rainforest. This area is also extremely humid, high‐yielding, and prone to disease; it is also home to different varieties of cocoa and different strains of the “national” cocoa variety than the region west of the Andes. Images were gathered at research stations and farms of varying sizes across these regions. The smallest farms had five to 10 trees, while the largest farms had 200,000 to 500,000 trees.

Data collection was guided in part by a ResNet18 CNN that we trained on images scraped from the internet and which was deployed using a Google Pixel 3a smartphone. This allowed us to identify and gather images that were misclassified by this initial prototype model. For example, we found that images of young cocoa leaves that were scraped from the internet were mostly infected with witches' broom disease. As a result, this initial model tended to classify young cocoa leaf images as having this disease, so we bolstered the dataset by gathering many images of healthy young cocoa trees and leaves.

This dataset consists of four classes: black pod rot (BPR; *Phytophthora palmivora*), witches' broom disease (WBD; *Moniliophthora perniciosa*), frosty pod rot (FPR; *Moniliophthora roreri*), and healthy cocoa. In this case, “healthy” cocoa constituted all trees not infected by disease and so included abiotic stressors such as damage from solar radiation or mechanical damage from overzealous pruning. A fifth class in this dataset was the “non‐cocoa” class, which was created by sampling randomly from the non‐cocoa images of the FAIGB dataset with a similar frequency to the other four classes. Cocoa images from the web‐scraped dataset were also added to the Ecuador dataset in the corresponding classes. In total, these five classes had the following number of images: BPR: 1752, FPR: 1907, WBD: 1620, Healthy: 1941, and NotCocoa: 1963. The frequencies of these classes, divided into disease categories diagnosed in the field and the corresponding image sources, are summarised in Table 1. All images not scraped from the web or sampled from the FAIGB dataset were taken in the field in Ecuador with either a Google Pixel 3a, Samsung Galaxy Xcover 4 (Seoul, South Korea), Samsung Galaxy J3, or an Olympus OMD‐EM4 camera (Tokyo, Japan). While most images from the field were taken by the authors, many were taken by additional volunteers. This use of a variety of cameras and photographers helped to control for the effect of camera and sensor design, different post‐image capture processing techniques applied by the devices, and any biases of the photographers.

**Table 1:** The frequency of images in the focal cocoa dataset by class and camera manufacturer/source.

Class^a^	Google	Olympus	Samsung	Vivo^b^	Web	Total
BPR	310	202	1092	81	67	1752
FPR	658	325	735	103	86	1907
Healthy	615	364	675	74	213	1941
WBD	326	674	577	0	43	1620
**Total**	1909	1565	3079	258	409	**7220**

^a^ Image classes include black pod rot (BPR, *Phytophthora* spp.), frosty pod rot (FPR, *Moniliophthora roreri*), healthy cocoa, and witches' broom disease (WBD, *Moniliophthora perniciosa*).

^b^ Images in the Vivo class came from the “Enfermedades cacao” dataset hosted by Kaggle (Serrano, 2019).

Smaller cocoa plantations were searched for disease in a grid pattern, while in larger plantations the search for disease was guided by the farmers. Images were labelled in the field as they were collected. In cases where a diagnosis was ambiguous, pods were dissected to confirm the diagnosis after taking photos and/or the advice of experienced people was sought.

In addition to capturing as many photos of diverse symptoms as possible, we also worked to include a variety of backgrounds as well as potentially misleading features that may confuse a CV model deployed in the field, such as machete damage, insect or small animal damage, and bird excrement on cocoa pods.

### Semi‐supervised training

Here we take the model training procedure developed in Sykes et al. (2025) and wrap it in a semi‐supervised learning loop as shown in Appendix S1.

**Figure 2:**
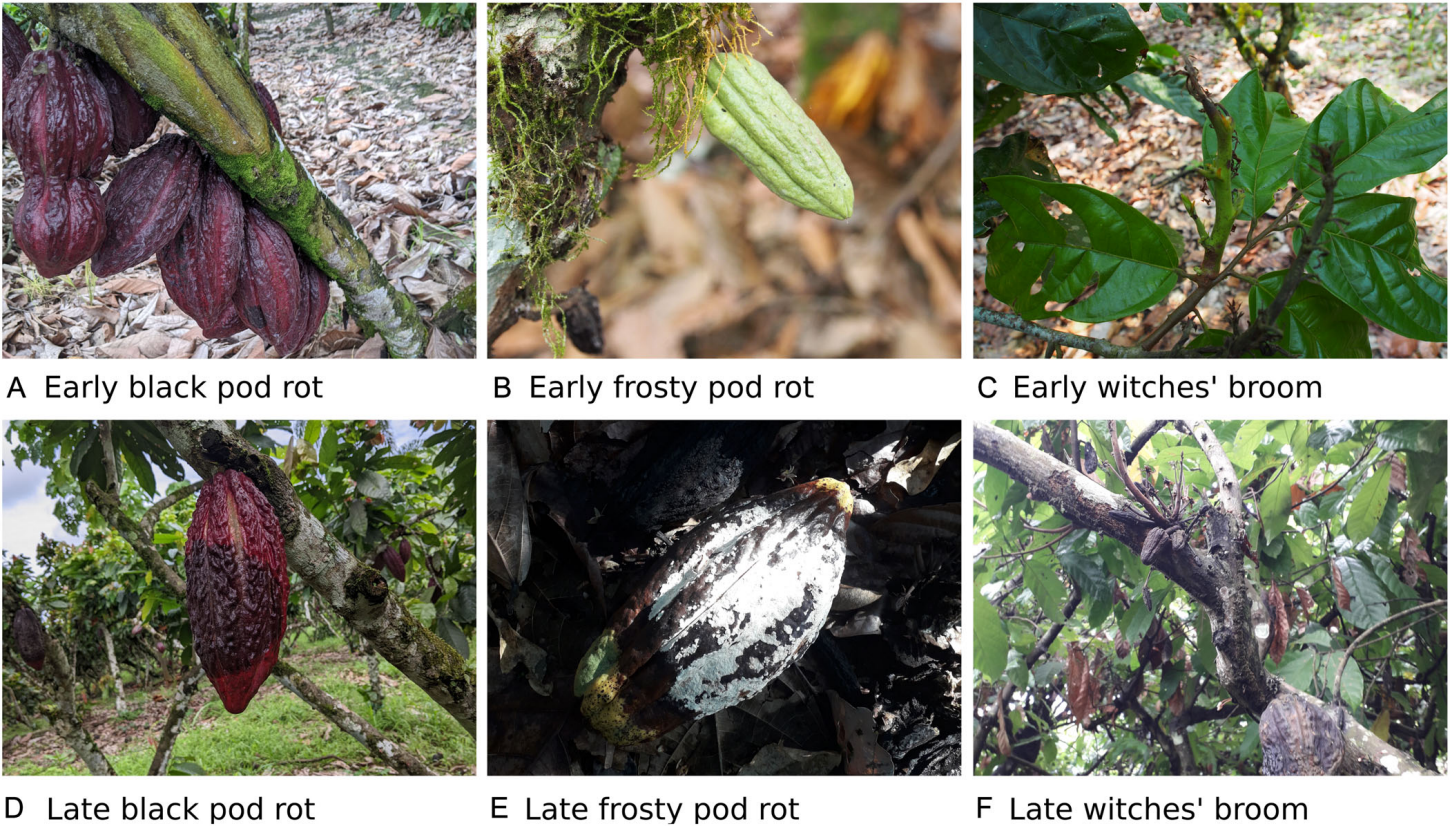
Visual progression of three cocoa diseases: black pod rot (A, D), frosty pod rot (B, E), and witches' broom disease (C, F). The examples show early and late symptoms, demonstrating the temporal discontinuity in disease symptom progression, especially in the case of frosty pod rot.

During data collection, the images were labelled as one of the four classes (BPR, FPR, Healthy, and WBD) and then labelled as “Easy” or “Difficult” to classify or diagnose or as “Unsure” if the classification was uncertain. This allowed for the semi‐supervised learning approach to be applied in cases where the images were considered difficult to classify or where human classification using only the image was unsure. Such images were added to the training data by the model only if they contained informative features. The iterative semi‐supervised learning process was allowed to continue until either no more images were relabelled or the validation F1 score of the model failed to increase after re‐training with the latest batch of relabelled images. Originally, the labels “Early” and “Late” stage disease development were to be used for this purpose, with the intention that the model would progressively learn to detect earlier and earlier disease symptoms. However, while this method may have worked for BPR, which presents with small brown external lesions that turn into large brown external lesions over time, it would be unrealistic to expect a CV model to understand how late‐stage FPR or WBD symptoms relate to early‐stage symptoms, as both of these diseases have an invisible intermediary stage that occurs within the pod or branch tissue (Figure 2). Taking FPR as an example, the almost imperceptible bump is symptomatic of initial infection (Figure 2B). This bump subsequently disappears as the pod continues to grow, seemingly in good health, while the inside is being destroyed by the fungus. The fungus then reappears on the outside of the pod (Figure 2E), engulfing it in irregular brown lesions and then with white mycelium. In developmental psychology and video‐based object tracking, the phenomenon that may allow a person or model to reason about the location or state of invisible objects is referred to as object permanence and has proved to be exceedingly difficult for neural networks to model (Shamsian et al., 2020). Additionally, early‐stage WBD can be just as easy to detect as late‐stage WBD. However, using the Easy and Difficult labels allowed for small or obscured but late symptoms to be sensibly labelled as Difficult, for early but obvious symptoms to be labelled as Easy, and for the apparently healthy side of a pod to be labelled as Unsure if it had a prominent lesion on the other side.

The disease state was labelled in the field but the difficulty label was applied in the lab. Upon visually reviewing each image, the Easy label was applied to images of pods or trees with clear and easily diagnosable symptoms; the Difficult label was applied if the disease state could be diagnosed with confidence but symptoms were small, distant, slightly obscured from view, or atypical; and the Unsure label was applied to images that could not be diagnosed by a human from the image. Such an image might not allow for diagnosis because: (1) the diagnosis in the field was only confirmed after taking the photo of the intact pod and subsequently dissecting it, (2) the visible symptoms used for diagnosis in the field were intentionally obscured from view to create images of this class, or (3) the symptoms were present and visually clear but cryptic. This method should allow for informative patterns found by the model in the Easy images to be reinforced as training continues, and it should prevent the model from overfitting to data that does not contain informative features. By encouraging such deductive reasoning, we aim to allow the CV model to learn features for disease detection that humans do not use, without forcing it to find spurious correlations.

### Dynamic focal loss

The original focal loss (FL), which like DFLoss is an extension of cross‐entropy loss, was designed to address the overwhelming class imbalance typical of one‐stage object detection models (Lin et al., 2017). FL adjusts the loss of observations according to a predefined class weight and the probability of an incorrect prediction derived from the model's output logit values. This function aims to penalise the model for attempting to lower loss by constantly classifying all images as the more frequent class, while also penalising the model for focusing its efforts on easy observations. This is the aim of DFLoss as well, but DFLoss also includes an empirical measure of difficulty in classification based on ground truth data and past classification attempts.

FL is based on the assumption that the softmax‐transformed logit values of a neural network will behave as a probability distribution for all possible classes of a given image. While this is valid pragmatically speaking, it is important to note that these are not probabilities derived from empirical frequency but are conditional probabilities based on the model's learned parameters and training data. As such, they can be treated as probabilities as long as we acknowledge that this remains valid only in the confined model training environment and does not extend to the real world.

The key difference between FL and DFLoss is an additional measurement of the difficulty of each observation that is made empirically and then combined with the probability of an incorrect prediction as defined in FL. Thus, DFLoss not only encourages a model to focus on observations for which its abstract measure of probabilities is most awry but, throughout training, it increasingly forces the model to focus on observations that it has objectively struggled to classify throughout training.

In DFLoss, a dictionary of uniform weights for each observation is generated and stored at the start of training. Beginning with the second epoch, when the model fails to classify an image correctly, the stored weight for that image is increased by a previously optimised amount (delta). For each batch of images, this weight is summed and applied to the loss value in place of the gamma smoothing value in FL, forcing the model to focus its efforts on classifying the difficult observations correctly, regardless of class. When the probability of an incorrect prediction approaches zero, the effect of this additional weight is lessened, and when an observation is classified correctly, DFLoss reverts to simple cross‐entropy loss. This second point is not true of FL. In DFLoss, an image may gain a higher weight during training because its informative features are less frequent in the training data or because those features are less apparent. By contrast, FL weights an image statically based on class and dynamically based on the pseudo‐probability distribution calculated by cross‐entropy relative to the label distribution. As such, DFLoss should achieve the same effect as FL for class imbalance without the need for an optimised class weight. We will test the effect of DFLoss with the unbalanced FAIGB dataset in future work. In the present work, we test the efficacy of DFLoss against cross‐entropy loss and a custom implementation of FL defined for a balanced multi‐class use cases, as shown below.

#### Defining dynamic focal loss

According to Lin et al. (2017), given the cross‐entropy loss (CE) for each observation in a batch, the probability of correctly classifying the target class (pt) is defined as

$$\mathrm{pt}=e^{-\mathrm{CE}}$$

The focal loss (FL) for an individual example, usually weighted by α (here we set α to 1, as we are not interested in class imbalance) and γ (focusing parameter), is defined as

$$\mathrm{FL}\left(\mathrm{pt}\right)=\alpha {\left(1-\mathrm{pt}\right)}^{\gamma }\mathrm{CE}$$

Finally, the FL averaged over a batch of N observations is calculated as

$$\mathrm{FL}=\frac{1}{N}\sum_{\left(i=1\right)}^{N}\alpha {\left(1-pt_{i}\right)}^{\gamma }CE_{i}$$

By contrast, in DFLoss, γ is a dynamically measured weight based on the difficulty that the model had predicting the class of a given observation in previous epochs. DFLoss is defined as

$$\mathrm{DFLoss}=\frac{1}{N}\sum_{\left\{i=1\right\}}^{N}\left(1+{\left(1-\mathrm{pt}\right)}^{\gamma_{i}}1_{\left\{\mathrm{pre}d_{i}\neq \;\mathrm{labe}l_{i}\right\}}\right)\mathrm{CE}\left(y_{i},\hat{y_{i}}\right)$$

$$\gamma_{i}=\sum_{\left\{j=1\right\}}^{N}w_{j}1_{\left\{\mathrm{pre}d_{j}\neq \mathrm{labe}l_{j}\right\}}$$

$$1_{\left\{\mathrm{pre}d_{i}\neq \mathrm{labe}l_{i}\right\}}=1\;\mathrm{if}\;\mathrm{pre}d_{i}\neq \mathrm{labe}l_{i},\;\mathrm{otherwise}\;0$$

With cross‐entropy loss defined as *CE*, the dynamic adjustment based on prediction correctness and weighting by γ is defined as follows:

- For each sample i, a unique weight $w_{i}$ is continually updated during training based on whether the prediction is correct or incorrect. Incorrect predictions result in an increased weight $w_{i}$, starting from 1 and incremented by δ for each incorrect prediction of the sample. δ should be optimised as a hyperparameter.
- The aggregated γ is the sum of weights $w_{i}$ for all samples in the batch that are incorrectly classified.

### Addition of a non‐cocoa class

In addition to the images of cocoa collected from Ecuador and scraped from the web, models here were also trained and tested with and without an additional “non‐cocoa” class. These 1963 images were randomly sampled from the FAIGB dataset of commonly grown arable crops and forestry species, both healthy and diseased, in roughly equal frequency to the other four classes. All cocoa images in the FAIGB dataset were manually filtered out of this subset. The objective of including this class was to prevent the model from overfitting to features that are not unique to cocoa or a specific cocoa disease. By exposing the model to such a wide variety of non‐cocoa plant images that are similar to those it may encounter in a real‐world application, the model is allowed to learn more robust correlations. For example, while bare soil does equate to an increased probability of BPR, because *Phytophthora* spp. is soil‐borne and splash‐dispersed, it is not inherently symptomatic of BPR. Similarly, an image showing the sky does not equate to FPR just because *Moniliophthora roreri* is wind‐dispersed and is typically found above 2 m in the canopy. This additional class should bolster the already robust cocoa dataset and help prevent the model from learning spurious correlations.

### Grad‐CAM analysis

In the field of deep learning, it is crucial not only to assess the accuracy of a model but also to evaluate how the model arrives at given conclusions. While traditional metrics like loss, accuracy, and F1 score provide quantitative assessments of model performance, they do not reveal whether the model is focusing on the correct features within an image to make its predictions. This understanding is especially important in contexts where interpretability and trust in the model's decisions are paramount, such as in medical imaging or agricultural applications where accurate disease identification can significantly impact production and management decisions.

**Figure 3:**
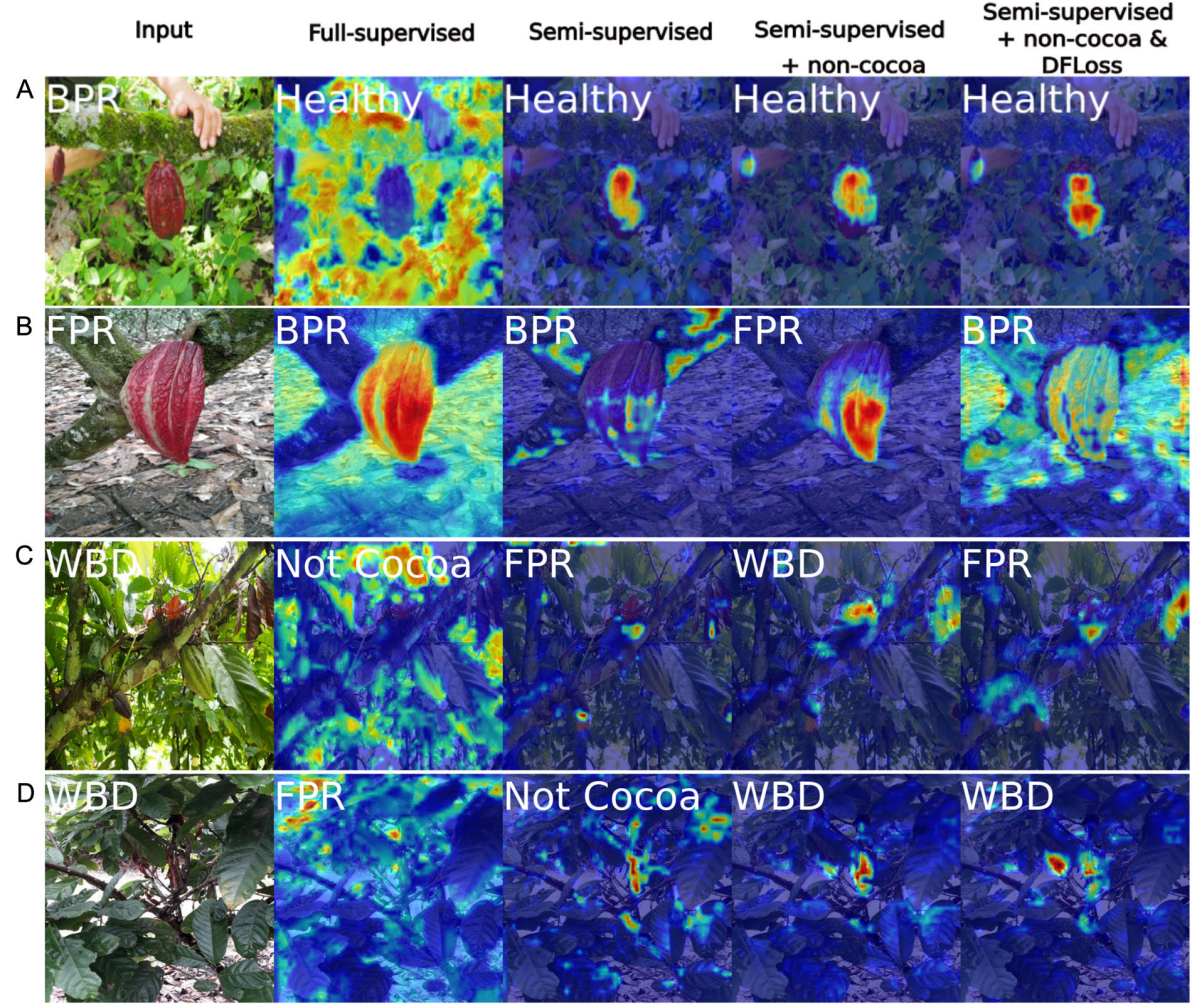
Example Grad‐CAM class activation maps with scores for PhytNet trained with four of the model training procedures shown in Table 2. The Grad‐CAM scores shown range from 0 to 1 and represent both how well each model classified the image and how well it focused its attention on relevant features. (A) Fully supervised: 0, semi‐supervised: 0.5, semi‐supervised+Non‐cocoa: 0.5, semi‐supervised+Non‐cocoa & DFLoss: 0.5; (B) 0.5, 0, 1, 0; (C) 0, 0, 1, 0; (D) 0, 0, 1, 1. For example, in the top image, all models classified the photo as healthy and focused their attention on healthy pod tissues but failed to detect the black pod rot lesion. BPR, black pod rot; FPR, frosty pod rot; Healthy, healthy cocoa; WBD, witches' broom disease.

In response to this, in addition to metrics like loss, accuracy, and F1 score, we used a qualitative Grad‐CAM score to compare models and training variants. This score was derived by manual visual assessment of class activation maps from Grad‐CAM (Figure 3) for each model and training procedure and then assigning a score of 0, 0.5, or 1 to each image. To produce the activation maps, an arbitrary number of images (in this case, 40) were randomly sampled from the Difficult cocoa images, which here also serves as a holdout test set. If a model correctly classified all images and focused its attention on relevant image features, it would score 1 averaged over the 40 images. However, if the model classifies an image correctly but fails to focus its attention appropriately, it will score 0 for that image as this would be a strong indicator of overfitting. If the model misclassifies an image but the classification and attention displayed appear sensible, it would score 0.5 for that image because it has not overfit, although it is a suboptimal classifier. For example, the image in Figure 3A was scored 0, 0.5, 0.5, 0.5. In this case, the fully supervised model scored 0 as it failed to correctly label the image or focus its attention on relevant features. However, the other three variants scored 0.5 as, although they failed to detect the very small BPR lesion at the base of the pod, they assigned a Healthy label because they focused their attention on the healthy tissue of the cocoa pod. The image in Figure 3B was scored 0.5, 0, 1, 0 because the fully supervised model focused its attention well but failed to classify the image as FPR, which was evident due to the obvious malformation of the pod. In contrast, the non‐cocoa variant was able to correctly classify the image and focus its attention appropriately and so scored 1 for that image. The Semi‐supervised and DFLoss‐trained models, however, misclassified the image and failed to focus their attention well, and so scored 0 for this image.

### Architecture choice

In this study, we evaluated the proposed training techniques using PhytNet and ResNet18. PhytNet, a lightweight CNN tailored for plant disease diagnosis, was developed to excel on noisy, plant‐specific datasets where computational resources are limited (Sykes et al., 2025). ResNet18, despite its age, remains a robust and efficient benchmark, widely used in state‐of‐the‐art hybrid models like DETR (Carion et al., 2020).

Prior work has extensively tested state‐of‐the‐art model architectures, including EfficientNet (Tan and Le, 2019) and ConvNeXt (Liu et al., 2022), finding that they tended to overfit on specialised datasets and offered no performance advantage over PhytNet or ResNet18 for tasks typical of agriculture and botany (Sykes et al., 2025). Rather than comparing a wide variety of model architectures, here we focus on techniques to improve the performance of architectures that have been proven to balance performance, interpretability, and computational efficiency, ensuring alignment with the constraints of agricultural applications.

## RESULTS

### Semi‐supervised learning and the non‐cocoa class

**Figure 4:**
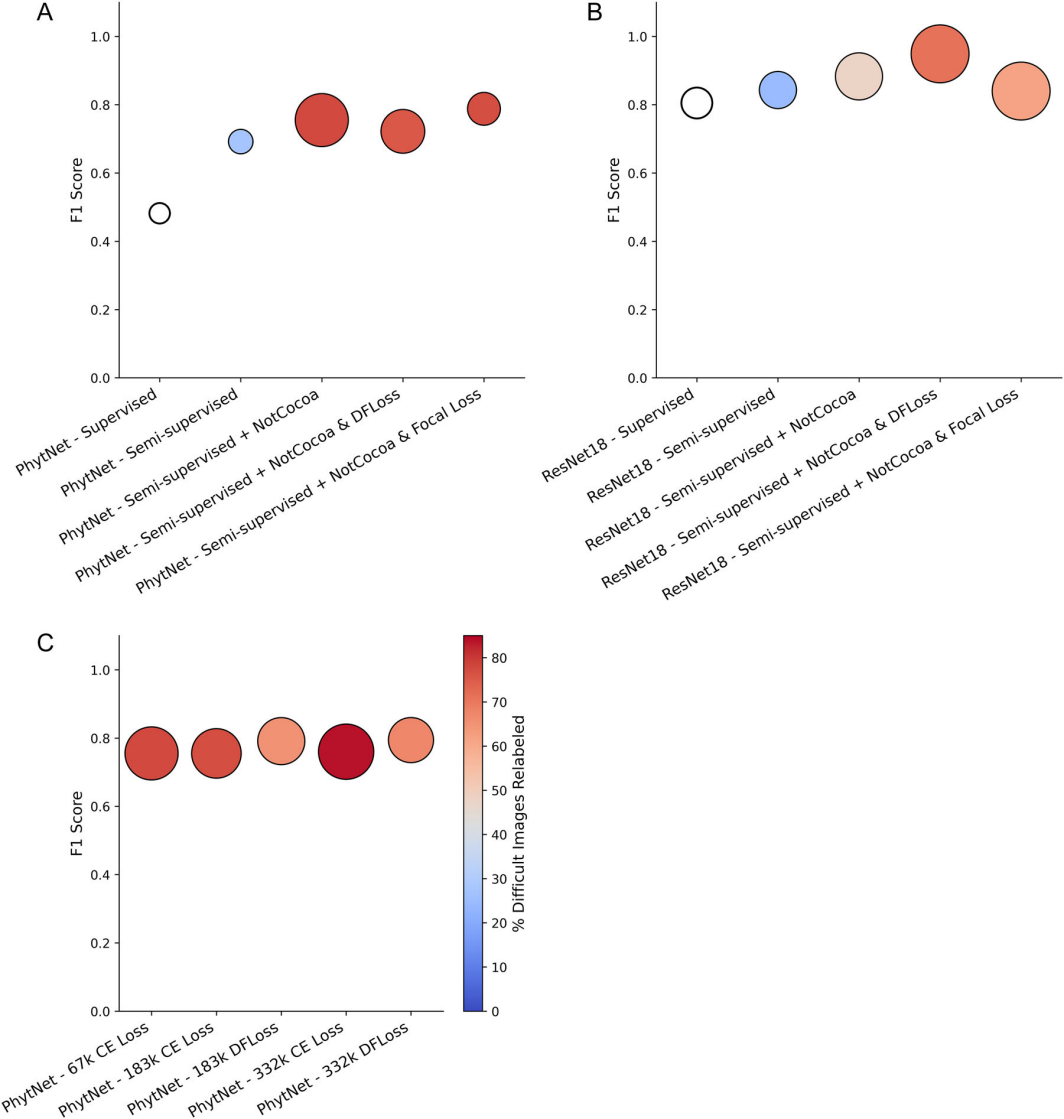
Comparison of model performance by validation F1 score, Grad‐CAM score, and percentage of difficult images relabelled during semi‐supervised training across multiple experiments. The Grad‐CAM score is shown by the size of the circles and the percentage of difficult images relabelled is shown by colour, according to the colour scale defined to the right of (C). Note that fully supervised models are shown as white, as no relabelling occurs during their training. (A) The PhytNet comparison shows the increase in F1 scores from supervised to semi‐supervised training, with both semi‐supervision and the addition of the non‐cocoa class showing marked improvements. (B) The ResNet18 comparison again demonstrates higher F1 scores under semi‐supervised training, with further notable gains from dynamic focal loss (DFLoss). (C) The PhytNet variants comparison highlights the absence of an effect from an increasing number of model parameters (67k, 183k, and 332k) and the deleterious effect of DFLoss on the percentage of difficult images relabelled.

For the cocoa image dataset, the semi‐supervised learning method increased the validation F1 score of PhytNet markedly from 0.482 to 0.692, while only slightly increasing the disparity between the training and validation scores (Table 2, Figure 4A). However, semi‐supervised learning did not increase the F1 scores uniformly across all classes, as BPR was already notably higher than FPR, Healthy, and WBD, which were increased by 20.9%, 27.9%, and 27.2%, respectively. Semi‐supervised learning also increased the Grad‐CAM score of PhytNet by 3.75%, and it increased the validation F1 score and the Grad‐CAM score of ResNet18 by 3.8% and 0.875, respectively.

**Table 2:** Performance metrics of PhytNet with four training procedure variants.

		Average		Per‐class training/validation F1 scores							
Variant	Metric	Train	Val.	BPR	FPR	Healthy	Not Cocoa	WBD	Difficult (%)^a^	Unsure (%)^a^	Grad‐CAM^b^
Supervised	Loss	1.222	1.210	0.582/0.654	0.352/0.375	0.451/0.463	N/A	0.380/0.418	N/A	N/A	0.088
	Accuracy	0.445	0.480								
	Precision	0.470	0.524								
	Recall	0.445	0.480								
	F1 score	0.449	0.482								
Semi‐supervised	Loss	0.597	0.849	0.770/0.725	0.671/0.584	0.808/0.742	N/A	0.776/0.690	27.54	30.98	0.125
	Accuracy	0.757	0.688								
	Precision	0.787	0.709								
	Recall	0.757	0.688								
	F1 score	0.763	0.692								
Semi‐supervised+NotCocoa	Loss	0.422	0.692	0.779/0.727	0.672/0.564	0.828/0.737	0.985/0.941	0.806/0.682	77.93	40.66	0.588
	Accuracy	0.834	0.756								
	Precision	0.838	0.760								
	Recall	0.834	0.756								
	F1 score	0.834	0.755								
Semi‐supervised+NotCocoa& DFLoss	Loss	0.522	0.708	0.774/0.714	0.556/0.375	0.815/0.714	0.931/0.980	0.381/0.632	75.57	43.51	0.4
	Accuracy	75.00	72.00								
	Precision	0.782	0.729								
	Recall	0.750	0.720								
	F1 score	0.750	0.722								
Semi‐supervised+NotCocoa& Focal Loss	Loss	0.407	0.579	0.788/0.8	0.706/0.619	0.873/0.735	0.982/0.939	0.696/0.8	77.32	56.95	0.225
	Accuracy	0.84	0.78								
	Precision	0.864	0.815								
	Recall	0.84	0.78								
	F1 score	0.841	0.788								

*Note*: BPR = black pod rot; DFLoss = dynamic focal loss; FPR = frosty pod rot; WBD = witches' broom disease.

^a^ Percentage of Difficult and Unsure images relabelled during semi‐supervised learning.

^b^ The Grad‐CAM score shows how well each model focused its attention on informative features with a range of 0–1.

With the exception of the DFLoss variant, ResNet18 overfits quite severely to this data (Figure 4B, Table 3). Here, all training loss values are one to three orders of magnitude smaller than the respective validation loss values. Additionally, the ResNet18 training F1 score for all variants is greater than 99%. While adding the non‐cocoa class resulted in validation F1 scores closer to parity with the training F1 score, such high training metric values are highly indicative of overfitting. Additionally, all but 0.2% of the increase associated with the addition of the non‐cocoa class in ResNet18 was due to the high per‐class validation F1 score (99%) that ResNet18 was able to achieve for this class, rather than an increase in the score of the other classes. Likewise, if we take the mean per‐class F1 score for the SemiSupervised + NotCocoa model for the BPR, FPR, Healthy, and WBD classes only (0.678), we see the addition of the non‐cocoa class decreased the mean validation F1 score by 1.4% relative to semi‐supervised alone (0.962). For both the ResNet and PhytNet models, however, the addition of the non‐cocoa class markedly increased the percentage of Difficult and Unsure images relabelled during training. This suggests that, despite this additional class causing a slight perturbation to the F1 scores for the easy images, it had a notable beneficial impact on the model's ability to generalise to unseen and more difficult cases. The superior validation F1 score (0.755), percent Difficult (77.93%) and Unsure (40.66%) images relabelled, and the Grad‐CAM score (0.588) for PhytNet SemiSupervised + NotCocoa show that both the semi‐supervised learning method and the addition of the non‐cocoa class greatly improved the model performance.

**Table 3:** Performance metrics of ResNet18 with four training procedure variants.

		Average		Per‐class training/validation F1 scores							
Variant	Metric	Train	Val.	BPR	FPR	Healthy	Not Cocoa	WBD	Difficult (%)^a^	Unsure (%)^a^	Grad‐CAM^b^
Supervised	Loss	0.015	0.667	0.997/0.795	0.995/0.751	0.996/0.828	N/A	0.996/0.847	N/A	N/A	0.2
	Accuracy	0.0.996	0.805								
	Precision	0.996	0.809								
	Recall	0.996	0.805								
	F1 score	0.996	0.805								
Semi‐supervised	Loss	0.003	0.774	0.999/0.838	0.999/0.778	0.999/0.870	N/A	1/0.883	24.36	11.73	0.288
	Accuracy	0.999	0.843								
	Precision	0.999	0.844								
	Recall	0.999	0.843								
	F1 score	0.999	0.843								
Semi‐supervised+NotCocoa	Loss	0.007	0.485	0.998/0.835	0.996/0.775	0.999/0.873	1/0.99	1/0.879	47.75	26.08	0.463
	Accuracy	0.999	0.883								
	Precision	0.999	0.890								
	Recall	0.999	0.883								
	F1 score	0.999	0.883								
Semi‐supervised+NotCocoa&DFLoss	Loss	0.00019	0.160	1/0.955	1/0.900	1/0.926	1/0.983	1/0.957	71.14	35.6	0.7
	Accuracy	1	00.95								
	Precision	1	0.951								
	Recall	1	0.95								
	F1 score	1	0.949								
Semi‐supervised+NotCocoa&Focal Loss	Loss	0.041	0.445	1/0.864	1/0.81	1/0.718	1/1	1/0.774	61.69	40.55	0.7
	Accuracy	1	0.84								
	Precision	1	0.843								
	Recall	1	0.84								
	F1 score	1	0.84								

*Note*: BPR = black pod rot; DFLoss = dynamic focal loss; FPR = frosty pod rot; WBD = witches' broom disease.

^a^ Percentage of Difficult and Unsure images relabelled during semi‐supervised learning.

^b^ The Grad‐CAM score shows how well each model focused its attention on informative features with a range of 0–1.

### Dynamic focal loss

The application of DFLoss to PhytNet appears to negatively impact all metrics, except for a 4% improvement in the non‐cocoa validation F1 score (Table 2). Additionally, it reduces the training metrics, bringing them much closer to the corresponding validation metrics, which suggests a reduction in overfitting. However, DFLoss hardly affected the percentage of Difficult and Unsure images that were relabelled, which remains high (76% and 44%, respectively) (Table 2). In sharp contrast, applying DFLoss to ResNet18 resulted in a remarkably strong performance across all metrics (Table 3). While the high training metrics and low training loss suggest some overfitting, the high percentage of Difficult images that were relabelled (71%), moderate percentage of Unsure images relabelled (36%), and high Grad‐CAM score (0.7) provide strong evidence that this model fit very well to the data. Further evidence of the absence of dataset memorisation comes from the FPR validation F1 score (90%), which is lower than all other classes, as expected due to the pervasive nature of FPR symptoms.

Given that DFLoss did not improve PhytNet results, we tested the hypothesis that DFLoss was able to improve results from ResNet18 but not from PhytNet because PhytNet was under‐parameterised for this task. To do this, we assessed the performance of two other PhytNet variants that performed well in the PhytNet optimisation sweep and had a greater number of trainable parameters; the optimised configuration values of these three PhytNet variants are shown in Table 4. The three models to be compared will hereafter be referred to as PhytNet67k (shown as PhytNet above), PhytNet183k, and PhytNet332k, referencing the number of trainable parameters in each model. In the initial sweep, the three models had validation F1 scores of 0.701, 0.693, and 0.699, respectively. PhytNet67k was originally chosen from this sweep because it had the highest validation F1 score, and the loss and F1 values plotted during training showed the least signs of overfitting.

**Table 4:** Comparison of parameters for different PhytNet configurations. All parameters were optimised for the validation F1 score in a WANDB optimisation sweep using a Bayesian process.

Parameter	PhytNet67k	PhytNet183k	PhytNet332k
beta1	0.9650	0.9671	0.9657
beta2	0.9816	0.9574	0.9908
Conv. channels 1	79	126	104
Conv. channels 2	107	91	109
Conv. channels 3	93	89	110
Image input size	415	371	350
Kernel size 1	5	5	5
Kernel size 2	1	1	7
Kernel size 3	7	17	13
Learning rate	0.000298	0.000967	0.000134
No. of conv. blocks 1	2	2	1
No. of conv. blocks 2	1	1	2
Output channels	6	7	9
No. of parameters	67,302	183,227	331,852
GFLOPS	0.6049	0.8591	1.188

*Note*: conv. = convolutional layer; GFLOPS = giga floating point operations.

The results of an experiment to compare three PhytNet variants trained with cross‐entropy loss and with DFLoss are shown in Figure 4C and Table 5. When applied to PhytNet183k, DFLoss increased the mean validation F1 score to 79% while decreasing the mean training F1 score to 79%, suggesting a reduction in overfitting. DFLoss also increased the FPR validation F1 score by 14% to 72%, which is the highest of any PhytNet variant; this is very advantageous for deployment as FPR is the disease of greatest concern in autonomous detection because it can remain asymptomatic until the latest stages of progression. However, DFLoss also increased the WBD training F1 score by 4% without affecting the WBD validation F1 score, decreased the percentage of Difficult images relabelled by 12%, and reduced the Grad‐CAM score by 5%. As such, the modest gains that are afforded to PhytNet183k by DFLoss appear to be the result of increased overfitting. PhytNet332k shows a similar pattern, with modest improvements in mean training and validation metrics that appear to show slight improvements in model fit at the cost of worsened performance in Difficult and Unsure images and Grad‐CAM score.

**Table 5:** Performance metrics of PhytNet model variants with and without dynamic focal loss (DFLoss) applied.

		Average		Per‐class training/validation F1 scores							
Variant	Metric	Train	Val.	BPR	FPR	Healthy	Not Cocoa	WBD	Difficult (%)^a^	Unsure (%)^a^	Grad‐CAM^b^
67k CE loss	Loss	0.422	0.692	77.9/72.7	67.2/56.4	82.8/73.7	98.5/94.1	80.6/68.2	77.93	40.66	0.588
	Accuracy	83.4	75.6								
	Precision	83.8	76.0								
	Recall	83.4	75.6								
	F1 score	83.4	75.5								
183k CE loss	Loss	0.4869	0.7104	76.6/77.2	65.2/57.9	77.4/70.6	97.4/91.5	77.9/71.4	77.23	47.27	0.513
	Accuracy	80.5	74.9								
	Precision	82.5	76.9								
	Recall	80.5	74.9								
	F1 score	80.8	75.5								
183k DFLoss	Loss	0.630	0.684	0.583/0.773	0.718/0.720	0.708/0.745	0.982/0.923	0.824/0.714	65.4	46.24	0.463
	Accuracy	79.00	79.00								
	Precision	0.802	0.804								
	Recall	0.790	0.790								
	F1 score	0.794	0.791								
332k CE loss	Loss	0.2503	0.7698	85.2/74.8	82.3/59.8	91.5/73.1	98.4/92.5	91.8/69.1	83.7	39.84	0.638
	Accuracy	90.9	76.0								
	Precision	91.4	77.5								
	Recall	90.9	76.0								
	F1 score	90.8	76.0								
332k DFLoss	Loss	0.447	0.589	0.867/0.765	0.718/0.649	0.773/0.776	0.985/0.982	0.909/0.609	67.08	23.0	0.425
	Accuracy	86.00	79.00								
	Precision	0.874	0.811								
	Recall	0.860	0.790								
	F1 score	0.860	0.794								

*Note*: BPR = black pod rot; CE loss = cross entropy loss; DFLoss = dynamic focal loss; FPR = frosty pod rot; WBD = witches' broom disease.

^a^ Percentage of Difficult and Unsure images relabelled during semi‐supervised learning.

^b^ The Grad‐CAM score shows how well each model focused its attention on informative features with a range of 0–1.

### Focal loss

The optimised value of FL gamma for PhytNet67k (1.0027) was close to 1, suggesting that the model performed optimally when the effect of FL was close to zero. Despite this, FL markedly improves disease‐specific validation F1 scores across the three disease classes while maintaining a high percentage of Difficult images relabelled (Table 2). However, the much‐elevated percentage of Unsure images relabelled (57%) and the very low Grad‐CAM score (0.225) represent strong evidence that FL achieved these F1 gains through overfitting. By contrast, in ResNet18, the optimised FL gamma value was 2.885, which suggests that FL had a beneficial impact on the validation F1 score during the sweep. However, FL decreased the validation F1 score in ResNet18 by 4% when evaluated after semi‐supervised training (Table 3). Although FL decreased the ResNet18 mean validation F1 score, it also increased the percentage of Difficult images that were relabelled (62%) to the second highest of any ResNet18 variant, behind that of DFLoss (71%). With FL, ResNet18 also maintained a modest percentage of Unsure images relabelled (40.55%) and increased the Grad‐CAM score so that it was equal to that of DFLoss, which had the highest score at 0.7. FL also increased the FPR validation F1 score to 81%, which is the second highest of any ResNet or PhytNet variant, behind ResNet18 with DFLoss (90%).

### PhytNet versus ResNet

While the highest validation F1 score for the best‐fit PhytNet variants (∼76%) is perhaps underwhelming, this model, like in the original paper (Sykes et al., 2025), shows almost no sign of overfitting. Additionally, PhytNet67k and PhytNet183k relabelled 78% of all Difficult images correctly, incorporating them into their training sets. This is ∼7% higher than the best‐performing ResNet variant. The similarity between the percentage of Difficult images relabelled and the Easy dataset F1 values further suggests that PhytNet is learning mostly genuine and broadly applicable patterns.

While the second‐best ResNet18 variant in terms of Difficult images relabelled (62%) performed well by this metric and in the Grad‐CAM score (0.7), there is a 16% difference between the mean training and validation F1 scores and a 22% difference between the mean validation F1 score and the percentage of Difficult images relabelled, which here serves as the holdout test set. However, with DFLoss applied, ResNet18 gives by far the best results except in the number of Difficult images relabelled and training loss. All training variants for both PhytNet and ResNet (perhaps with the exception of PhytNet with FL) show a relatively low number of Unsure images relabelled. This is encouraging, as many of these images do not contain symptoms detectable by humans or CV models, meaning that performing with too high of a value for this metric would strongly suggest overfitting. However, none of these factors appears to be an issue for ResNet18 trained with semi‐supervised learning, the additional non‐cocoa class, and DFLoss. This model appears to perform exceptionally well on most metrics.

**Figure 5:**
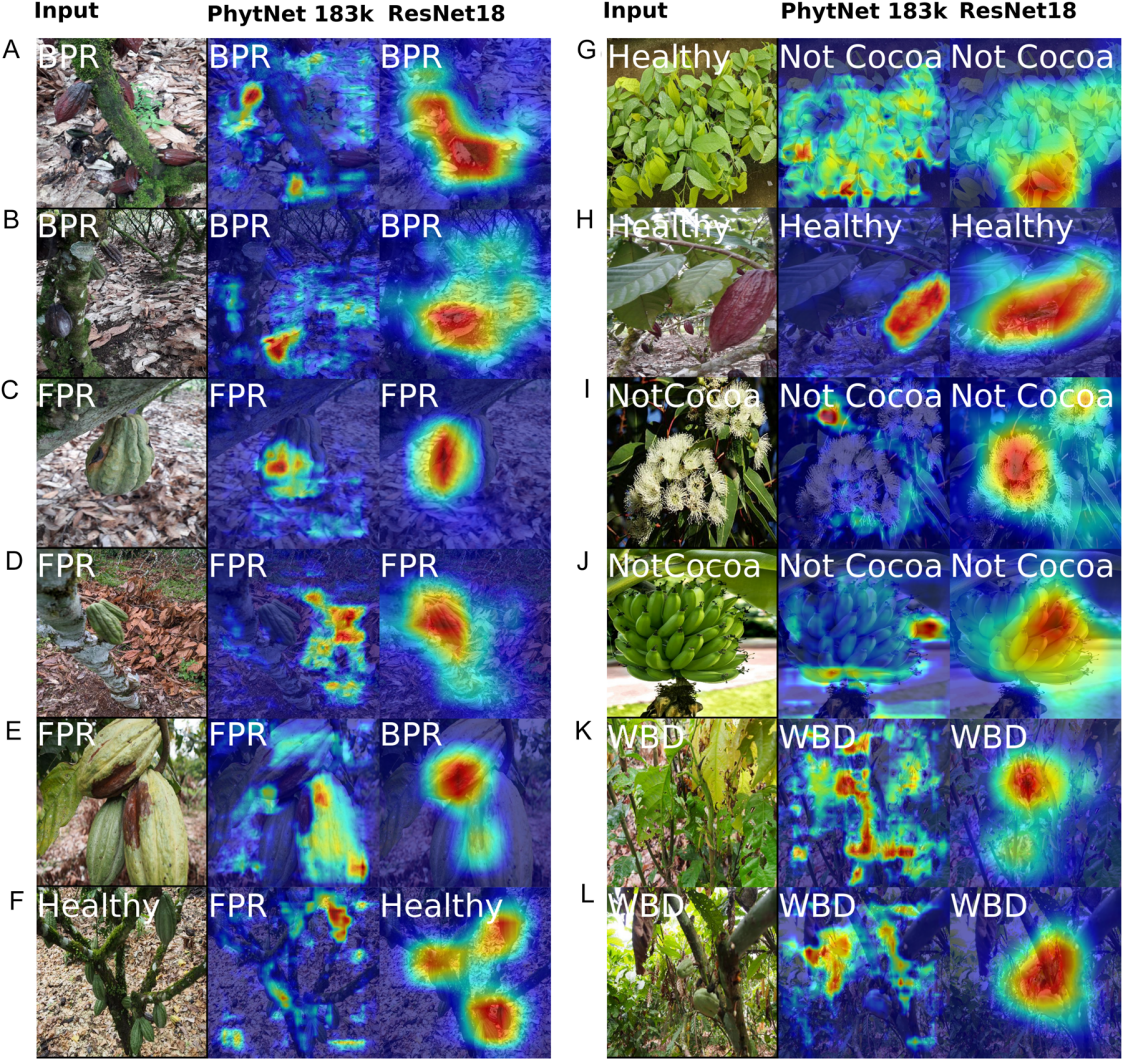
Grad‐CAM visualisation of the class activation maps generated by the best‐performing PhytNet183k and ResNet18 models. PhytNet183k was trained using semi‐supervised learning, incorporating an additional cocoa class and cross‐entropy loss, while ResNet18 employed semi‐supervised learning with the additional cocoa class and dynamic focal loss. The maps highlight the regions that were most influential in disease classification, showcasing differences in focus and localisation accuracy between the two models.

Both PhytNet and ResNet18 tended to perform well at attending to informative features; however, there were also cases where one or both models failed (Figure 5). For example, (1) both models are shown to correctly classify an image as BPR while attending to the bare soil (Figure 5A). This is a textbook sign of overfitting to correlative features in the dataset. (2) PhytNet is shown to correctly classify an image as FPR while attending more to the healthy tissue than the lesion (Figure 5C), while ResNet18, perhaps understandably, misclassifies this image as BPR while attending well to the necrotic tissue. (3) Both models correctly classify the image in Figure 5D, while PhytNet focuses its attention on irrelevant leaf litter in the background. This is a strong sign of dataset memorisation, a form of overfitting that will lead to complete failure upon deployment in the real world. (4) Both models are shown to attend well to the young nursery cocoa plants (Figure 5F), yet both fail to classify these plants as healthy cocoa.

In addition to comparing models by GFLOPs and number of trainable parameters, we also looked at more practical metrics (Table 6), which included the frames per second (FPS) on a GPU and a CPU as well as the GPU compute utilisation and peak VRAM utilisation. With the image input size optimised for the present dataset (3,375,375), we calculated the GFLOPS of ResNet18 to be 5.24, which far exceeds that of PhytNet183k (GFLOPS: 0.86). When evaluated on an Nvidia H100 GPU (Nvidia, Santa Clara, California, USA) with a batch size of 100 images, the mean FPS of ResNet18 was 33 fewer than PhytNet183k and 15 FPS fewer when evaluated on a CPU. These are notable differences if one aims to scale up deployment. GPU compute utilisation was 4.96% greater in PhytNet183k than ResNet18, while peak VRAM utilisation was 5% lower in PhytNet (Table 6). Note that the optimised image input size of PhytNet183k for the present dataset (3,371,371) is almost identical to that of ResNet18.

**Table 6:** Runtime performance metrics for two neural network models.^a^

Model	No. of parameters	GFLOPS	FPS (GPU)	FPS (CPU)	GPU utilised (%)	Peak VRAM usage (MB)
ResNet18	11,689,512	5.24	963	85	35.65	280.07
PhytNet183K	183,227	0.86	996	100	40.61	266.46

*Note*: FPS = frames per second; GFLOPS = giga floating point operations.

^a^ The GPU used was an Nvidia H100 80GB and the CPU was an AMD EPYC 7643 48‐core processor. Batch size: 100.

## DISCUSSION

In this study, we explored various training strategies and model enhancements to improve the performance of deep neural networks. We focus on ResNet18 and PhytNet in the detection of diseases in cocoa plants, while incorporating a discussion on runtime performance metrics. The overarching findings reveal significant advancements in model accuracy, generalisation capabilities, and interpretability through the incorporation of semi‐supervised learning, the addition of a non‐cocoa class, and the implementation of specialised loss functions—FL and DFLoss. Additionally, the use of Grad‐CAM for qualitative assessment provided deeper insights into model behaviour, offering a more nuanced understanding of where these models focus their attention and how this relates to their prediction accuracy.

### Efficacy of semi‐supervised learning

The semi‐supervised learning approach used here markedly improved all performance metrics for both PhytNet and ResNet18. Many previous studies have used semi‐supervised learning to incorporate both labelled and unlabelled data into the training process to effectively enhance the model's learning capability, particularly in scenarios where labelled data are unavailable or expensive to obtain (Van Engelen and Hoos, 2020). However, here we show that it can be used to allow models to learn from correctly labelled images with symptoms that were difficult to detect with the human eye or invisible to humans, without forcing overfitting. While this form of semi‐supervised learning still necessitates the laborious and costly task of data labelling, we show here that this effort is well spent in training models that generalise better to the real world than those trained with full supervision. This effect is apparent in the qualitative Grad‐CAM scores, which showed a 37.5% and 87.5% increase for PhytNet and ResNet18, respectively, with this approach. Crucially, in PhytNet67k, semi‐supervised learning increased the FPR validation score by 20.9%. This improvement to the FPR F1 score is of critical importance to the application of these models in the field, as FPR poses a significant risk if it spreads to Brazilian or African crops (Bowers et al., 2001; Ploetz, 2016) and it is the most difficult of the three diseases to detect (Guillaumin et al., 2010).

### Inclusion of the non‐cocoa class

With the inclusion of the non‐cocoa class, we aimed to prevent overfitting to irrelevant features by exposing the model to a wider variety of features that it is likely to encounter in the real world. A concerted effort was made during data collection to include features that would help prevent overfitting. The inclusion of the non‐cocoa class extended this effort to include images of a great variety of plants and disease symptoms, which include features that are distinct from but could be mistaken for those relevant to the detection of cocoa disease. For example, *Phytophthora* spp., which causes BPR, infects dozens of other crop species (Kroon et al., 2012), many of which are included here in the non‐cocoa class. The inclusion of this class improved all performance metrics for both models and did so to a greater degree than any other additional method employed here. Moreover, the dramatic increase in both the number of Difficult and Unsure images relabelled and the improved Grad‐CAM scores, coupled with a reduced disparity in training versus validation values, shows that the inclusion of this class had a marked beneficial effect in the reduction of overfitting.

### Focal loss and dynamic focal loss

While standard FL was developed to address the problem of massively imbalanced classes in binary data, it is also intended to help focus model attention more on observations that the model struggles to classify. Similarly, DFLoss was developed and implemented here to address the challenge of learning from difficult or less frequent observations in a multi‐class scenario and should also help with cases of class imbalance. This latter feature was not tested here, as the present data are well‐balanced, but will be tested in future work. The varied results observed here in testing DFLoss across different models indicate that its effectiveness may be model‐specific. While DFLoss greatly improved performance in ResNet18 without increasing overfitting, in PhytNet DFLoss only appeared to increase overfitting. This behaviour is likely due to the fact that PhytNet is specifically designed to be appropriately parameterised for a given dataset. So here, after running the PhytNet optimisation sweep with cross‐entropy loss, PhytNet lacked the spare capacity to make use of DFLoss. By contrast, the present results show that, without DFLoss, ResNet18 is clearly over‐parameterised to this dataset and so had the spare capacity to do well with DFLoss. To test this explanation in future work, we will evaluate PhytNet's performance using DFLoss prior to the optimisation sweep.

If it is not possible to avoid the use of an over‐parameterised model, DFLoss offers potential benefits in improving model fit by utilising the spare model capacity. Potential reasons why DFLoss would decrease overfitting in ResNet18 include: (1) that it is acting as a form of regularisation by preventing the model from overly focusing on the more frequent or easily recognisable features, or (2) given that training accuracy was 99.9% without DFLoss and so the model could hardly fit more to the training data, DFLoss might have caused the model to learn the genuine patterns of some images before it learned too many false patterns.

FL appears to increase the validation metrics of PhytNet slightly, but it did not affect the percentage of Difficult images relabelled, it greatly increased the percentage of Unsure images relabelled, and it greatly reduced the Grad‐CAM score. Based on these results, FL only increased overfitting in PhytNet without any improvement in its ability to classify images. By contrast, FL appears to decrease overfitting in ResNet18, although to a lesser extent than DFLoss. FL led to a marked improvement in the percentage of Difficult images relabelled and the joint highest Grad‐CAM score of any model variant.

As with model pruning, rather than using tools like FL to combat overfitting retrospectively, it should be preferable to begin with a model that is appropriately scaled to the dataset at hand (Liu et al., 2018). While a great deal of research effort has been applied to model pruning and quantisation without loss of performance, some loss appears inevitable (Wu et al., 2019; Solodskikh et al., 2023). Similarly, the results presented here for FL further vindicate the use of appropriately scaled models and the avoidance of hacking model behaviour in convoluted ways; however, the effectiveness of DFLoss with ResNet18 appears to contradict this narrative. To investigate this matter further, we should develop new means of detecting and explaining overfitting in ResNet18 with DFLoss. This is because the very low training loss suggests it is still overfitting, even if all other metrics show it is also learning genuine patterns in the data very well. Additional work is also required to improve the performance of PhytNet, which shows great potential in its superior ability to classify Difficult images over ResNet18 with far fewer parameters and with greater computation efficiency.

### Grad‐CAM analysis

The use of the Grad‐CAM score for quantifying the inherently qualitative analysis of reviewing class activation maps adds a novel dimension to the present evaluation of model performance. While the subjective nature of such analyses can pose difficulties in objectivity and reproducibility, they can be highly informative when paired with other rigorous quantitative analyses. The visual inspection of data and results is vital in data analysis, but objective comparisons become difficult in the huge datasets typical of analyses with deep neural networks. The simple scoring system employed here worked well in allowing for the subjective analysis of model attention through class activation maps and a meaningful comparison of models. For example, when paired with the decrease in the percentage of Difficult images relabelled, the decreased Grad‐CAM score allows for the differentiation between PhytNet183k with and without DFLoss (Table 5).

### Model selection

Considering the wealth of performance data presented here, the selection of the “best” model is far from simple. There are three main criteria by which we will judge model performance: (1) summary statistics such as accuracy and F1 score, (2) model attention and ability to generalise, and (3) runtime speed and compute requirements.

#### Summary statistics

While metrics like F1 score and loss are highly informative, model choice is not a simple matter of choosing the highest validation F1 score and lowest loss. Rather, we must consider these metrics in context to provide meaningful insights into the fit of the model and its behaviour. For example, when trained with cross‐entropy loss, ResNet18 scored an impressive 99.9% training F1 score and 88.3% validation F1 score. However, with FL added, the same model scored 4% lower validation F1 score but relabelled 14% more Difficult images and 14% more Unsure images, i.e., it performed markedly worse on easy images but much better with difficult and unsure images. Additionally, both PhytNet and ResNet18 are shown to focus on bare soil at the base of a tree while correctly classifying the image as BPR (Figure 5A). Cocoa pods touching bare soil like this are at extremely high risk of BPR as the causative agent is soil‐borne and dispersed by splashing rain drops (Nkeng et al., 2017), yet this is not a reliable signal. Such insights cannot be gained from summary statistics alone.

While all PhytNet variants scored with roughly equal validation F1 scores (74.5–79.4%), PhytNet183k with cross‐entropy loss scored one of the highest for validation F1 values (75.5%), with the second lowest training F1 score (80.8%). This, in conjunction with the modest Grad‐CAM score, suggests that, of the PhytNet variants, PhytNet183k fit best to this data without overfitting. However, its Grad‐CAM score was exceeded by as much as 15% by other PhytNet variants, again highlighting the complexity of model choice. Additionally, the metrics of PhytNet are generally overshadowed by those of ResNet18 with DFLoss, although this is not true of any of the other ResNet18 variants, which had quite variable performance metrics and showed many signs of overfitting.

#### Model attention and ability to generalise

In comparing the Grad‐CAM scores, we see that only one of the PhytNet variants (67k: 0.588, 183k: 0.513, 332k: 0.663) performed comparably to the best ResNet18 variants (0.7). However, we also see that an improved Grad‐CAM score does not directly correlate with reduced overfitting, as we might expect. The Grad‐CAM scores and Figure 5 show that despite ResNet18 showing evidence of overfitting in its loss values, it does well in many cases at focusing its attention on relevant features. This suggests that, while ResNet18 may have overfit to the data, it also learned many relevant features; however, PhytNet appears to have learned many of these same relevant features with two to three orders of magnitude fewer trainable parameters. While PhytNet332k had the second‐highest Grad‐CAM score, it also showed signs of overfitting by the large disparities between the training and validation metrics. However, this disparity was reduced by DFLoss. Additionally, despite being 45% larger than PhytNet183k, PhytNet332k only scored 0.3–0.5% greater validation F1 score than PhytNet67k. For these reasons, PhytNet183k is likely the best of the PhytNet variants and will be used below for drawing comparisons on runtime speed and compute requirements against ResNet18. However, one might well choose PhytNet332k due to the high percentage of Difficult images that it relabelled (84%) or its high Grad‐CAM scores (64%) with cross‐entropy loss.

#### Runtime speed and compute requirements

While there are many metrics by which we can compare the runtime speed and compute requirements of two models, by far the most important for real‐world deployment are FPS and peak VRAM utilisation. While the difference in VRAM utilisation between the PhytNet183k and ResNet18 is negligible, the difference in FPS on a CPU and GPU is more pronounced. Scaled up, these differences amount to an extra 118,800 images per hour with PhytNet183k over ResNet18 on a GPU. As such, if the chosen model is to be run on low‐powered hardware or if large scale is required, then PhytNet183k may be preferable. This, coupled with PhytNet's much superior performance on difficult test images and relatively high Grad‐CAM attention scores, suggests that PhytNet has the potential to out‐compete ResNet18 for this dataset. However, with the addition of DFLoss, ResNet18 is shown to be the likely choice.

### Conclusions

Through semi‐supervised learning, specialised loss functions, and the inclusion of a non‐cocoa class, we achieved improvements in model accuracy, generalisation, and interpretability. Notably, PhytNet demonstrated superior performance on difficult test images with superior computational efficiency, making it particularly suitable for deployment on low‐powered hardware. The Grad‐CAM analysis added a valuable qualitative dimension, revealing where models focused their attention and providing insights into their decision‐making processes.

While we selected ResNet18 and PhytNet for their balance of performance and computational efficiency, this study did not include the latest transformer‐based models such as Real‐time DETR (Lv et al., 2024), which, although significantly larger in number of parameters, offer fast runtime speeds and could provide a fruitful area for future work. Additionally, our reliance on fully labelled data for semi‐supervised learning remains a limitation, as it is both labour‐intensive and costly. Further research should explore methods to reduce this dependency and investigate the generalisability of these models across more diverse datasets.

By addressing these challenges, the continued development of efficient and robust models like PhytNet can play a critical role in real‐world disease monitoring and intervention, enabling scalable solutions for sustainable food production.

## AUTHOR CONTRIBUTIONS

J.R.S. conceived of this study, read and summarised the relevant literature, gathered the data, conducted the analyses, and wrote the first draft of the manuscript. K.J.D. and D.W.F. had substantial input in experimental and data analysis design, continually reviewed and edited the manuscript, and approved the final manuscript before submission and publication. All authors approved the final version of the manuscript.

## Supporting information


**Appendix S1.** Semi‐supervised learning loop.

## Data Availability

All data are made available via the Open Science Framework. The cocoa image data is available at https://osf.io/2fw6g, and the FAIGB web‐scraped dataset of crop disease images is available at https://osf.io/nuafh. All code necessary to reproduce these results is available on GitHub (https://github.com/jrsykes/CocoaReader/tree/main/CocoaNet/PhytNet_Cocoa).

## Appendix 1 Latitude and longitude of data collection locations.

**Table:**

Name	Latitude	Longitude
Riviera cacao	−2.083064	−79.28616
Hacienda San Jose	−1.888611	−79.489074
Naranjal	−2.697633	−79.643309
Near Riviera	−2.064715	−79.244245
INIAP ‐ Litoral Sur	−2.256634	−79.644175
Riviera cacao ‐ associate	−2.677806	−79.65014
Riviera cacao Tambo	−1.980887	−79.258232
INIAP ‐ Pichiligue	−1.075552	−79.492659
Patricia Pilar	−0.539016	−79.36657
Calceta	−0.850179	−80.180915
INIAP ‐ Amazon	−0.340658	−76.874867
Ceracita	−2.330835	−80.270115
