# Supplementary material for: Improving computer vision for plant pathology through advanced training techniques
Source: Appl Plant Sci. 2025 Jun 7;13(3):e70010. doi: 10.1002/aps3.70010 (PMC12188622; doi:10.1002/aps3.70010)
Supplement: Supplementary file 1 — Appendix S1. Semi‐supervised learning loop. [file APS3-13-e70010-s001.docx]

## **Appendix S1.** Semi-supervised learning loop.

train(model)

**for** ToLabelDataSet in [DifficultImages, UnsureImages] **do**

**while** nRelabeledImages > 0 **do**

previous F1 = eval(model)

**for** image in ToLabelDataSet **do**

classify image

**if** prediction = label **then**

label image & add to train data

**end if**

**end for**

train(model)

new F1 = eval(model)

**if** new F1 < previous F1 then

end training

**end if**

**end while**

**end for**
